# Advances in mortality forecasting: introduction

**DOI:** 10.1186/s41118-018-0045-7

**Published:** 2018-12-19

**Authors:** Fanny Janssen

**Affiliations:** 1 Population Research Centre, Faculty of Spatial Sciences, University of Groningen, Groningen, The Netherlands; 2 Netherlands Interdisciplinary Demographic Institute, The Hague, The Netherlands

## General introduction

Mortality forecasts are essential for predicting the future extent of population ageing, and for determining the sustainability of pension schemes and social security systems. They are also useful in setting life insurance premiums, and in helping governments plan for the changing needs of their societies for health care and other services (European Commission 2009). Nowadays, the societal importance of accurate mortality forecasts is greater than ever before. As a strategy for dealing with rapid population ageing, recent pension reforms in a number of low-mortality countries have made an explicit link between the retirement age and/or retirement payments and past and future anticipated mortality and life expectancy values (Carone et al. 2016; OECD 2016). Because of the large and increasing societal relevance of accurate mortality forecasts, the field of mortality forecasting is growing and advancing.

To respond to the challenges associated with population ageing, numerous models for mortality modelling and forecasting have been developed in recent decades (Tabeau 2001; Booth and Tickle 2008; Girosi and King 2008; Cairns et al. 2011a; Shang et al. 2011). The majority of the currently existing mortality forecasting methods, including the mortality forecasting methods used by statistical offices in Europe, are extrapolative (Booth and Tickle 2008; Stoeldraijer et al. 2013). These extrapolative approaches make use of the regularity observed in both age patterns and mortality trends over time (Booth and Tickle 2008), and are considered more objective, easier to apply and more likely to result in accurate forecasts than the other two types of approaches to mortality forecasting: explanation approaches (mortality forecasting by cause of death or with an explanatory model) and expectation approaches (based on the opinions of experts) (Booth and Tickle 2008).

For many years, the Lee-Carter mortality projection methodology (Lee and Carter 1992) has been the benchmark extrapolative mortality forecasting method (Booth and Tickle 2008; Shang et al. 2011; Stoeldraijer et al. 2013). The Lee-Carter age-period mortality model decomposes age-specific mortality (logged) over a certain time period for a single population into the overall time trend, the age pattern of mortality and the age-specific differences in the extent of the overall time change. An error term is included to capture age-period effects that are not captured by the model. Subsequently, mortality is forecasted by extrapolating the overall time trend using standard time-series procedures (Lee and Carter 1992). Among the clear advantages of the Lee-Carter methodology are that it comprises a simple stochastic model with only one time-varying parameter; it performs relatively well when past trends have been linear; and it is able to forecast a changing age pattern of mortality (Booth and Tickle 2008).

Many variants and extensions of and alternatives to the Lee-Carter model have been developed since its inception. For example, a number of alternative estimation procedures have been proposed to improve the model fit (Lee and Miller 2001, Booth et al. 2002, Brouhns et al. 2002, Currie et al. 2004; Hatzopoulos and Haberman 2009). The functional data approach was developed as an alternative to the Lee-Carter model. This approach includes higher order principal components and nonparametric smoothing (Hyndman and Ullah 2007), and provides a more realistic future age pattern of mortality. Also other procedures (Ishii 2008; Li et al. 2013) have been proposed to cope with the much criticised assumption of a fixed age pattern of change in the Lee-Carter model (Booth and Tickle 2008). The Cairns-Blake-Dowd (CBD) models (Cairns et al. 2006, Cairns et al. 2009, Li and O’Hare 2017) were proposed to better capture mortality at ages 55 and over. These CBD models model the logit of the death probabilities at older ages as a linear or quadratic function of age, thereby treating the intercept and slope parameters across years as stochastic processes (Villegas et al. 2018). In addition, to improve the forecast performance of the Lee-Carter model particularly when past mortality trends have been non-linear, the inclusion of a cohort parameter (Renshaw and Haberman 2006; Cairns et al. 2009; Plat 2009; Cairns et al. 2011b; Reither et al. 2011) and different approaches to detect and deal with structural change (Booth et al. 2002; Coelho and Nunes 2011; van Berkum et al. 2016) have been proposed and implemented.

In addition, other more general advances in mortality forecasting have been made that deal with a major challenge that arises when using extrapolative approaches: i.e., identifying the underlying long-term mortality trend that can truly serve as the basis for extrapolation (Janssen and Kunst 2007). This process is not just a purely mathematical exercise, but requires a careful analysis of past national and international mortality trends and its determinants (Janssen and Kunst 2007).

One important related advance in mortality forecasting is the (further) development of coherent multi-population forecasting methods. Coherent multi-population mortality projections have been developed to avoid unrealistic crossovers or divergence in future mortality between countries or sexes, which can result from applying mortality projection models to single populations/countries [hereafter referred to as individual mortality forecasting] (Li and Lee 2005; Enchev et al. 2017). The main idea behind coherent forecasting is that mortality forecasts for populations with similar mortality developments will not diverge radically, but that structural differences will remain (for instance, consistently higher mortality for men than for women) (Hyndman et al. 2013). Coherent forecasts also lead to improved predictive power because structural mortality trends can be better predicted when trends in similar populations (e.g., countries) are used for the identification of the most likely future developments (Janssen and Kunst 2007). The extension of the Lee-Carter model to multi-populations by Li and Lee (2005) has been the trend setter. In subsequent years, additional coherent forecasting methods were developed that were based on the Lee-Carter structure (Li and Hardy 2011; Russolillo et al. 2011; Li 2013; Wan et al. 2013; Wang and Yang 2013; Zhou et al., 2014; Kleinow 2015), as well as on the age-period-cohort structure (Cairns et al. 2011b; Dowd et al. 2011; Jarner and Kryger 2011; Börger and Aleksic 2014) and the functional data approach (Hyndman et al. 2013; Shang and Hyndman 2017). Recent evaluation studies have demonstrated that these coherent mortality forecasting models are indeed superior to individual mortality forecasting models (e.g. Li et al. 2017; Shang 2016; Shair et al. 2017). Coherent multi-population mortality forecasting models have mainly been applied to a group of countries (see as well Torri and Vaupel 2012), both sexes in a population (see as well Raftery et al. 2014 and Hatzopoulos and Haberman 2013), or regions within a country (see as well Bergeron-Boucher et al. 2017 and Alexander et al. 2017). However, these forecasting models have rarely been applied to socio-economic groups (Villegas and Haberman 2014; van Baal et al. 2016; Cairns et al. 2016), even though there are substantial differences in mortality by socio-economic group (e.g. Murtin et al. 2017).

Another significant related advance is the inclusion of information about the determinants of past trends in the mortality forecast. Although using an objective extrapolative mortality forecasting approach has clear advantages, it does not take into account important information about the determinants of past trends and important (temporal) deviations from the overall linear mortality trend (Janssen and Kunst 2007; Olshansky et al. 2009). Consequently, the use of general extrapolative mortality forecasting methods, especially in a situation where past mortality trends have been nonlinear, can result in different outcomes when different historical periods are chosen (Janssen and Kunst 2007; Stoeldraijer et al. 2013), and the effect of this explicit choice of the historical period can be greater than the effect of the type of extrapolative forecasting method chosen (Stoeldraijer et al. 2013). Moreover, the use of general extrapolative mortality forecasting methods can also generate unlikely future patterns when temporal increases or declines in mortality have occurred, as these are automatically considered structural in extrapolative mortality forecasting approaches (Janssen et al. 2013). To overcome these issues, several recent studies have incorporated epidemiological information into mortality forecasting, predominantly by means of information on smoking and/or obesity (Pampel 2005; Bongaarts 2006; Wang and Preston 2009; King and Soneji 2011; Preston et al. 2012; Janssen et al. 2013; French and O'Hare, 2014). These studies employed different approaches. Many of the studies that focused on the USA included detailed lifestyle information from the national health survey in the forecasting model (Wang and Preston 2009; King and Soneji 2011; Preston et al. 2012). French and O'Hare (2014) included aggregate covariates on health behaviour (e.g. alcohol and tobacco consumption aged 15 and over) in the Girosi and King Bayesian hierarchical model (see King and Soneji 2011). Bongaarts (2006) and Janssen and Kunst (2007) focused on forecasting the more linear past trends in non-smoking-related mortality, rather than all-cause mortality. Pampel (2005) forecasted sex differences in all-cause mortality by combining a forecast of the sex ratio in smoking-related mortality—using a regression model expressing the relationship between smoking prevalence and smoking-related mortality 25 years later—with the presumed changes in the sex ratio in non-smoking-related causes of death. Janssen et al. (2013) combined the forecast of smoking-related mortality by means of age-period-cohort modelling with the forecast of non-smoking-related mortality by means of conventional extrapolative mortality forecasting techniques. Recently, Bongaarts (2014) showed that using such a novel approach is better than directly performing cause-specific mortality projections—i.e., than merely using an explanatory approach—as doing so takes advantage of the predictability of the discontinuity in mortality trends as a result of a behavioural cause, which is absent for regular causes of death. Janssen et al. (2013) combined the inclusion of the smoking epidemic in mortality forecasting with internationally coherent mortality forecasting.

Other important advances in mortality forecasting that have been discussed in the recent literature include (i) the use of different measures beyond age-specific mortality rates for the mortality forecast, such as the use of rates of mortality improvement, either applied to individual mortality forecasting (Haberman and Renshaw 2012; Haberman and Renshaw 2013; Mitchell et al. 2013) or to coherent multi-population mortality forecasting (Schinzinger et al. 2016; Bohk-Ewald and Rau 2017); (ii) the inclusion of output measures beyond life expectancy at birth, such as lifespan disparity (Bohk-Ewald et al. 2017); (iii) the use of multiple forecasts combined rather than a single forecast (Shang 2012; Kontis et al. 2017); (iv) the use of Bayesian probabilistic mortality forecasting instead of general stochastic/probabilistic mortality forecasting to make statements about what is not known, conditional on what is known, either applied to individual forecasting (Czado et al. 2005; Pedroza 2006; Kogure and Kurachi 2010; Wiśniowski et al. 2015; Wong et al. 2017; Hilton et al. 2018) or to coherent multi-population mortality forecasting (Cairns et al. 2011b; Raftery et al. 2013; Antonio et al. 2015; Bohk-Ewald and Rau 2017); and (v) the validation of the model used for mortality forecasting by means of in-sample forecasting (= backtesting) (e.g. Cairns et al. 2011a; Hyndman and Athanasopoulos 2018).

## The thematic issue “Advances in mortality forecasting”

The seven papers in the current thematic issue on “Advances in mortality forecasting” refer to many of the abovementioned recent advances in mortality forecasting, but they also propose additional advances in the field of mortality forecasting. These papers can be divided into three types. The first three papers—Shang and Haberman (2018), Barboutsos et al. (2018) and Bergeron-Boucher et al. (2018)—focus on the development, application and further refinement of more advanced mortality forecasting approaches for low-mortality countries. The fourth and fifth papers—Stoeldraijer et al. (2018) and Wilson (2018)—also address mortality forecasting in the context of low-mortality countries, but focus on questions that arise when dealing with mortality forecasts on a more regular basis. The final two papers—Fazle Rabbi and Mazzuco (2018) and Diaz and Debón (2018)—look at contexts in which mortality modelling is already a daunting task, and mortality forecasting is especially challenging, namely countries with much more volatile past mortality trends and more limited data availability.

The paper by Han Li Shang and Steven Haberman (Australian National University) addresses the recent trend towards the use of multiple forecasts combined rather than of a single forecast. Although this approach has long been popular in the fields of management science, psychology and statistics (Clemen 1989), it did not receive much attention in the demographic literature prior to the mid-1990s (e.g. Ahlburg 1998). For mortality forecasting, the contribution of Kontis et al. 2017 is noteworthy. They projected future life expectancy in 35 industrialised countries using a Bayesian ensemble of models, all of which—weighted according to their predictive power—contributed to the final projections. In the current thematic issue, Shang and Haberman analyse the effects of determining a set of statistically superior models from a predefined list of models, based on the model’s past out-of-sample performance, and subsequently applying equal weights to the selected superior models. Their approach was motivated by the work of Samuels and Sekkel (2017) and Hansen et al. (2011), and was applied to national and sub-national mortality data for Japanese men and women aged 60 and over. They showed that the proposed averaging approach is among the top-performing methods, when its point and interval forecast accuracy is compared with that of 17 time-series extrapolation methods (Renshaw-Haberman models, the Cairns-Blake-Dowd models, the Lee-Carter models and the functional time-series models). The Plat model performed best at the national level for both sexes and at the subnational level for women; the multilevel functional time-series method and the product-ratio method performed best at the subnational level for men. They also found that the proposed model averaging/-trimming method results in a smaller forecast error than the existing model averaging method, which assigns different weights to all models based on the model’s accuracy. The authors therefore recommend using the proposed trimming method to obtain forecasts outcomes that are robust to model misspecification.

Anastasios Bardoutsos et al. (University of Groningen, the Netherlands) focus in their paper on the inclusion of mortality delay and mortality compression in mortality projections. Although the shift of the age-at-death distribution to older ages and the decline in the variability in the age at death have been identified as the key dynamics shaping past mortality trends in low-mortality countries (e.g. Janssen and de Beer, n.d. (submitted)), they have seldom been included in mortality projections. Notable exceptions are Bongaarts (2005), Terblanche (2015) and Basellini et al. (2016). However, these approaches can only be applied to the adult population. In their contribution to the thematic issue, Bardoutsos et al. compare the model properties and the in-sample and out-of-sample projection performance of a new parametric mortality model that captures delay and compression of mortality across all ages (the CoDe model) (de Beer and Janssen 2016; de Beer et al. 2017) with the Lee-Carter model for France, Japan and the USA using data from 1960 to 2014. In making this comparison, the authors examined many different outcome measures (age-specific death rates, age-at-death distribution, life expectancy at birth, and the modal age at death), in line with the recent recommendation by Bohk-Ewald et al. (2017). Bardoutsos et al. find that whereas the LC model consistently projects a slowdown of mortality delay, and, consequently, a slowdown of increases in life expectancy at birth, the CoDe model can project a continuation of delay, and thus a steady increase in life expectancy. According to the authors, accounting for delay and compression is necessary for developing more robust and accurate projections of the age pattern of mortality. Bardoutsos et al. also emphasise that the CoDe model can be used as a diagnostic tool, and could be further extended to take into account lifestyle factors, the mortality experiences of other populations or socio-economic differences.

The paper by Marie-Pier Bergeron-Boucher et al. (University of Southern Denmark) looks at another important advance in mortality forecasting: coherent/multi-population forecasting. Specifically, the authors propose the use of a sex-ratio approach to coherently forecast mortality for men and women. Previous multi-population forecasting models (Li and Lee 2005; Hyndman et al. 2013; Shang 2016; Shang and Hyndman 2017) generally forecast a common mortality trend among all the populations and the population-specific deviation from this common trend. Similar to Raftery et al. (2014) and Pascariu et al. (2018), Bergeron-Boucher et al., in addition, take into account that females are likely to maintain lower mortality than males due to biological differences. Also, Bergeron-Boucher et al. control for the fact that differences between age groups exist in the trends in sex differences in mortality. The approach developed by Bergeron-Boucher et al. first forecasts female mortality by age, and then forecasts the sex ratio of mortality rates before and after age 45 to obtain the forecast of male mortality. They apply their approach to five prior sex-independent models for 18 industrialised low-mortality countries and compare the resulting out-of-sample forecasts with the out-of-sample forecasts of 5 sex-coherent models. They conclude that their sex-ratio approach leads to more accurate forecasts of the recent male mortality trends, without losing accuracy in the forecast for women. The authors acknowledge that if done properly, taking into account explanatory variables—such as the sex difference in health-related behaviours like smoking and alcohol—would represent an additional step forward.

The two additional contributions on low-mortality countries address important questions that arise when dealing with mortality forecasts on a more regular basis.

Lenny Stoeldraijer et al. (Statistics Netherlands) focus on the problem of choosing the appropriate jump-off rates, or the mortality rates used as the starting values of the actual mortality forecast. These rates are either those observed in the last year(s) or those estimated by the underlying mortality model. This issue has seldom been addressed in the previous scientific literature, and a general approach to choosing the jump-off rates is currently lacking in practice. Dealing with this problem is, however, important, as it is encountered in the majority of forecasting models because of a mismatch between the observed and predicted trends. The authors evaluate six different jump-off rate options, and examined their effects on the robustness and accuracy of a Lee-Carter forecast of life expectancy at age 65 for eight low-mortality European countries. They showed that the choice of the jump-off rates clearly influenced the accuracy and robustness of the mortality forecast, albeit in different ways. For most countries, using the observed values in the last year as the jump-off rates resulted in the most accurate forecast, whereas using an average of observed values over more years as the jump-off rates generated the most robust forecast. According to the authors, which strategy is the best for matching mortality forecasts to the most recently observed data depends on the goal of the forecast, the country-specific past mortality trends observed and the model fit. Especially in light of the increased importance of having regularly updated forecasts to inform pension reform measures, it is essential that the best possible jump-off rate is chosen.

In his contribution to the thematic issue, Tom Wilson (Charles Darwin University, Australia) highlights the importance of employing not just national mortality forecasts, but sub-national mortality forecasts. He also addresses the difficulties involved in transferring sub-national mortality forecasting methods to regions smaller than the state or province level, noting, for example, that the time-series of the input data for these regions may not be as long. More specifically, he evaluated the suitability of different methods for sub-state regional mortality forecasting for practising demographers and forecast users in government and business. He performed such an evaluation for Australia divided into 88 sub-state regions. Drawing on data from 2001 to 2016, he used the 2001–2006 data to generate within-sample forecasts for 2006–2011 and 2011–2016. In addition to assessing the accuracy, plausibility and smoothness of the mortality age profiles, he evaluated the input data requirements, the ease of calculation and the ease of assumption setting and scenario creation. After providing a review of the different approaches to regional mortality forecasting—including the application of models developed for single national populations to every region, coherent mortality models and relational mortality models—he evaluated eight fairly simple sub-national mortality forecasting models that have been implemented by statistical agencies or researchers in recent years. His analysis showed that National Death Rates, SMR Scaling and Rate Ratio Scaling are among the less suitable methods, while the remaining five methods are more suitable. Wilson observed that of these five remaining methods, the Broad Age SMR Scaling and the Broad Age Rate Ratio Scaling are easy to implement, the TOPALS method is more difficult to implement and the more complex Brass Relational method and Mortality Surface method produce the smoothest mortality age profiles and highly plausible death rates. His contribution illustrates the importance of carefully evaluating existing regional mortality forecasting methods in situations in which data for a shorter time period only are available. His research also points to the importance of using criteria in addition to accuracy when evaluating mortality forecasting methods, in line with the recommendation made by Cairns et al. 2011a.

The issue of having time-series data on mortality for a short period only was also addressed by Ahbad Mohammad Fazle Rabbi and Stefano Mazzuco (University of Padova, Italy) in their contribution on mortality forecasting in Central and Eastern Europe (CEE). Compared to the rest of Europe, the CEE countries have less available time-series data, higher mortality and more volatile past mortality trends. Because of these differences, the CEE countries have rarely been the focus of mortality forecasting papers in Europe, as the overwhelming focus on low-mortality countries in the current thematic issue illustrates. The paper by Bohk and Rau (2015) is among the few studies that have looked at mortality forecasting in CEE, and used the mortality trends of other selected countries to forecast changes in long-term mortality trends for Hungary and Russia. In their contribution to the current thematic issue, Fazle Rabbi and Mazzuco evaluated for nine CEE countries the performance of seven different variants of the Lee-Carter method, the Li-Lee coherent mortality forecast and the UN Bayesian hierarchical forecast method. Except for the weighted version of the Hyndman and Ullah (2007) method, all seven Lee-Carter variants resulted in future life expectancy that is lower than current life expectancy values, at least for Belarus, Russia and Ukraine. Their analysis showed that the coherent Li-Lee methodology was not useful because of the diverging trend of mortality over the fitting period. The use of the Bayesian method resulted in a better forecast than the use of some of the extrapolative methods, but it also produced greater uncertainty. After observing that the existing mortality forecasting methods performed poorly when applied to these CEE countries, the authors emphasise the need to develop a new flexible forecasting technique specifically geared to the features of past mortality trends in Central and Eastern Europe, and made some recommendations for how this might be done.

Daunting as well is the challenge of mortality forecasting for countries for which only abridged life tables by sex are available, such as Colombia. The contribution by Gisou Díaz and Ana Debón (University of Tolima, Colombia; University of Valencia, Spain) described these difficulties. Whereas the Human Mortality Database is an important source of very detailed and high-quality mortality information for low-mortality countries (Barbieri et al. 2015), mortality data for Latin America are collected in the Latin American Human Mortality Database (Urdinola and Queiroz 2017) and the most detailed life table information comes from abridged life tables. Applying current mortality modelling methods and mortality forecasting methods in a situation in which abridged life tables are the most important and the most detailed sources of information can be daunting. Díaz and Debón systematically evaluated the performance of seven variants of the Lee-Carter (LC) method using abridged life tables for the 1973–2005 period for Colombia, and the StMoMo package in R (Villegas et al. 2018). They demonstrated the difficulties involved in using different age-period-cohort (APC) models with the limited data, and in dealing with the high mortality among young adult men resulting from homicides, assaults or other violent acts. They found that the fit of the three models that in fact converged (the LC method, the LC method with two terms (LC2) and a simple APC method) was, in general, higher among women than among men. The LC2 outperformed the LC method, as it better captured the mortality hump among young adult men, while the simple APC method performed worst. In their discussion, the authors outline the implications of insurance companies failing to take into account the mortality hump among Colombian men.

## Conclusion

In sum, this thematic issue on “Advances in mortality forecasting” not only provides an overview of important recent advances in mortality forecasting and the current available advanced mortality forecasting approaches, it also introduces the reader to three novel and/or refined approaches for mortality forecasting in low-mortality countries. The sex-ratio approach proposed by Bergeron-Boucher et al. (2018) represents an alternative approach to coherently forecasting mortality for men and women. The novel mortality projection model developed by Barboutsos et al. (2018) explicitly takes into account the two dynamics underlying past mortality trends (mortality delay and mortality compression). Moreover, Shang and Haberman (2018)—inspired by earlier work—proposed the further refinement of the selection of mortality forecasting models when forecasting using multiple mortality forecasts combined instead of a single mortality forecast.

The current thematic issue also highlights the notion that the research questions and forecasting issues that are important in practice differ from those that are important in academic circles (see as well Gaille 2012). Stoeldraijer et al. (2018) emphasised that in order to obtain accurate and robust mortality forecasting outcomes, the jump-off rates must be chosen carefully. Wilson (2018) identified important practical issues that can arise when forecasting mortality at the sub-state level, and emphasised the role of additional evaluation criteria, such as input data requirements and the ease of assumption setting and scenario creation.

In addition, the papers in this thematic issue clearly demonstrate that the current extrapolative mortality forecasting techniques are not always easily applicable beyond low-mortality countries with regular past mortality trends. Fazle Rabbi and Mazzuco (2018) illustrated this for nine Central and Eastern European countries with volatile past mortality trends, whereas Diaz and Debón (2018) did so for Colombia using less detailed mortality data, and in a context of high homicide mortality among men.

Thus, while the current thematic issue gives important insights into recent advances in mortality forecasting, there seems to be a clear need for further innovations. These might include (i) the development of mortality forecasting models that can be applied beyond low-mortality countries with regular past mortality trends; (ii) efforts to take into account important research questions and forecasting issues from within the profession; (iii) the systematic incorporation of lifestyle factors into current mortality forecasting approaches; and (iv) efforts to improve mortality forecasting at the sub-national level—not only by region but by socio-economic group.
